# Understanding the Shared E-scooter Travels in Austin, TX

**DOI:** 10.3390/ijgi9020135

**Published:** 2020-02-24

**Authors:** Junfeng Jiao, Shunhua Bai

**Affiliations:** Urban Information Lab, The University of Texas at Austin, Austin, TX 78712, USA

**Keywords:** e-scooter, urban environment, travel pattern, shared micro-mobility

## Abstract

This paper investigated the travel patterns of 1.7 million shared E-scooter trips from April 2018 to February 2019 in Austin, TX. There were more than 6000 active E-scooters in operation each month, generating over 150,000 trips and covered approximately 117,000 miles. During this period, the average travel distance and operation time of E-scooter trips were 0.77 miles and 7.55 min, respectively. We further identified two E-scooter usage hotspots in the city (Downtown Austin and the University of Texas campus). The spatial analysis showed that more trips originated from Downtown Austin than were completed, while the opposite was true for the UT campus. We also investigated the relationship between the number of E-scooter trips and the surrounding environments. The results show that areas with higher population density and more residents with higher education were correlated with more E-scooter trips. A shorter distance to the city center, the presence of transit stations, better street connectivity, and more compact land use were also associated with increased E scooter usage in Austin, TX. Surprisingly, the proportion of young residents within a neighbourhood was negatively correlated with E-scooter usage.

## 1. Introduction

Shared, dockless E-scooters (e.g., Lime and Bird scooters, E-scooters from here on out) are the newest form of shared mobility services. They provide users with a fun, flexible way to fulfill their short-distance trips, and as a result, they have become extremely popular in many US cities (e.g., Austin, Chicago, Dallas, Denver, and Los Angeles) in recent years. E-scooter companies claim they provide a convenient, sustainable mode of transportation to help customers solve their first/last mile problem. However, there are very few empirical studies to back up any of these arguments. Moreover, public health scholars have even suggested the potential of E-scooter operation increasing carbon emissions [1].

Because of their convenience, flexibility, and pleasant riding experience, E-scooters have successfully satisfied the huge demand-supply gap in the market and have become very popular in many US cities (e.g., Austin, Chicago, Dallas, Denver, Los Angeles, etc.) in a matter of one year. Lime researched transit usage in New York City and argued that the existence of transit deserts [2] and income disparity were two major causes of declining transit ridership [3]. As a partial solution, Lime proposed using micro-mobility such as E-scooters to cover intermediate distances to and from transit stops.

E-scooters have created controversy for hosting cities in regard to irresponsible parking on sidewalks and in green spaces [4]. Due to the lack of dedicated road space for E-scooters, riders often use sidewalks or bicycle lanes to ride and park, impeding traffic of other modes. San Francisco’s Department of Public Works has accused E-scooter companies of contributing to illegal scooter parking in public spaces. Seattle, another bike-share-friendly city, even halted the operation of E-scooters altogether due to safety concerns. To preserve transportation flexibility and avoid potential conflicts, E-scooter operators and cities began implementing geofencing techniques to regulate future E-scooter usage. ‘Geofencing’ is a location-based technique that establishes a virtual boundary in an actual geographic area [5]. Researchers in Shanghai, China, found that geofencing policy could satisfy over 90% of total parking demand and reduce inappropriate parking behaviors [6]. San Jose, California, also considered establishing geofences to protect sidewalks from E-scooters. In response, the E-scooter company ‘Bird’ released a built-in geofencing tool called “GovTech Platform,” which reminds users to ride responsibly and park legally [7].

As E-scooters are relatively new to us, cities and transportation researchers are still at the early stage of discovering how E-scooter trips are distributed and how urban environments might relate to them. To expand the research boundary and to offer more empirical evidence, we investigated the spatial-temporal patterns of shared E-scooters in Austin, TX, via GIS spatial analysis. Then, we fit a negative binomial model to explore the relationship between E-scooter usage and some key built environments. The paper includes five sections. After the introduction, the second section provides a literature review of previous empirical E-scooter studies and dockless bike-sharing ones in retrospect. Then, we present data and analyses in the third section. Section 4 summarized the key findings from spatial-temporal analysis and statistical modeling. Finally, in the last section, we concluded the study by discussing the practical contribution of this paper and future research direction.

## 2. Literature Review

Previous studies directly focusing on E-scooters are emerging but are still very limited at the current stage. This is mainly for two reasons: first, since E-scooter operation is new and available only in a few large US cities, its influence has not yet piqued public curiosity; and second, most E-scooter datasets are generally unavailable to academia [7,8]. Still, researchers are starting to tease out the social impacts of E-scooters from various aspects. Hollingsworth et al. simulated the environmental burdens of manufacturing and operating E-scooters and found out that E-scooters generated less net air pollution during their life cycle [1]. Fang et al. examined several hundred photographs of parked E-scooters in San Jose and found that users frequently parked their devices on sidewalks, impeding pedestrian traffic [9]. Another study in Los Angles conducted by Allem and Majmundar in 2019 analyzed E-scooter’s social media posts and concluded that E-scooter promotions did not place adequate emphasis on the use of protective gear, a key to ensuring public safety [10].

By the completion of this study, there have only been two empirical analyses of E-scooters and built environments published, to our knowledge. McKenzie compared the spatiotemporal usage patterns of E-scooters and station-based bike-sharing in Washington D.C. and found out that E-scooter resembled spontaneous bike-sharing for leisure purposes more than the membership usage at a regular basis [11]. In a more recent study in Indianapolis, IN, Mathew and his team used GIS spatial analysis on the local E-scooter trip dataset and identified downtown and the university campus as two geographic hotspots of E-scooter traffic in the city [12]. In their report, they also found that people tended to ride E-scooters in the afternoon rather than the morning, implying that E-scooters were not used for the morning commute there. Limited empirical research on E-scooter operation in cities limits this field of transportation scholarship. Therefore, in the following section, we expanded our literature review to include related studies of dockless bikes. We expect to contrast their findings with ours later in this paper.

Dockless bike-sharing began satisfying personal travel demand before E-scooters by allowing users to locate and unlock vehicles through digital smartphone applications and pay online at the conclusion of a trip [13]. As of 2018, dockless bike-sharing systems had been introduced to 28 US cities and the number continues to grow [14]. Compared to station-based bike-sharing, dockless bikes provide more freedom and convenience to users. As a result, it would naturally reveal more information about users’ travel patterns [15–18]. In Singapore, researchers identified several factors that promoted the usage of dockless bikes, including compact land use, high accessibility to public transportation, and supportive cycling infrastructure [19]. Li et al. surveyed dockless bike users in Jiangsu Province, China, and found that most riders cycled short distances in urban centers where higher levels of education resources and employment were concentrated. The survey also found that income and education levels were positively associated with the usage of free-floating bike-sharing [20]. Gu et al. conducted a nation-wide empirical analysis on dockless biking behavior in China and concluded that the majority of riders were young and highly educated citizens satisfying commuting demands [21]. A Seattle study confirmed a similar circumstance in the US, such that dockless bike vehicle distribution favored socio-economically advantaged neighborhoods [22]. A research group in Singapore found the temporal and spatial concentrations of dockless bikes were mainly affected by the surrounding built environments such as residential density and street connectivity [23]. In addition to spatial-temporal characteristics, they also found a substantial discrepancy in biking time and travel distance on weekdays as opposed to weekends. Given that dockless bikes and E-scooters share many commonalities in terms of vehicle size, personal travel demand, and dockless technology, we expect to find similar results in the E-scooter study.

## 3. Data and Analyses

### 3.1. Data

As one of the major tech hubs in the US, Austin has a long history of accommodating various shared micro-mobility modes such as docked and dockless bikes and E-scooters. As of February 2019, there are 10 licensed operators with over 15,000 authorized dockless E-scooters in Austin [24]. To respond to the major influx of E-scooters, Austin announced the Dockless Mobility Project, which delineated the principles, rights and report mechanisms for all shared small vehicle mobility systems [25]. To increase safety, the Austin Transportation Department (ATD) allows operators to remotely disable a malfunctioning vehicle. To better manage parking issues, ATD now requires that all E-scooters must park in designated parking zones, defined as “hard surface within the landscape/furniture zone of a sidewalk with at least 3-foot pedestrian clear-zone” [25].

To better understand E-Scooter usage in Austin, this study gathered 11 months of dockless vehicle trip data (April 2018 to February 2019) from ATD. Each record in the dataset represented one trip made by dockless vehicles (i.e., E-scooters and bikes) with key trip information including OD (origin-destination) location, date, beginning and end time, trip duration, and trip distance. To protect user privacy, the City of Austin technology office spatially aggregated the specific OD coordinates to a hexagonal grid in which each hexagon is 0.023 square mile and has an edge length of 500 ft. After removing bike trips, this dataset included approximately 1.74 million E-scooter trip records and a sum of nearly 1.3 million miles traveled. To better appreciate this number, we can imagine Austinites “scooting” around the Earth’s equator roughly 52 times from 2018 to 2019.

To grasp the demographic/socio-economic background of where people rode E-scooters in Austin, we selected five commonly used measurements in previous travel behavior and built environment empirical studies [26,27], including population density, male/female ratio, percentage of young population, household income, and the percentage of people with bachelor’s degree or higher. The original data were acquired from the U.S. Census Bureau at the Census block group level. The measurements of the built environment included the location relative to the city core, the street connectivity nearby, the proximity to bus stops or light rail stations, and the land-use layout. We calculated all scores of the built environment measurements in ArcGIS 10.7 software. A detailed description of the measurements is provided in Section 3.3.

### 3.2. Temporal Analysis

The study first analyzed the monthly trip count, total vehicle miles traveled, average trip distance, and average operation time. Table 1 shows the descriptive statistics of E-scooter usage by month from April 2018 to February 2019. During this period, May 2018 had the lowest trip count, with over 5000 trips generated by 370 vehicles. Comparably, October 2018 had the highest, with over 241,000 trips generated by more than 7000 vehicles. The average trip distance in October was 0.77 miles, with an average operation time of 7.55 min.

**Figure 1.**
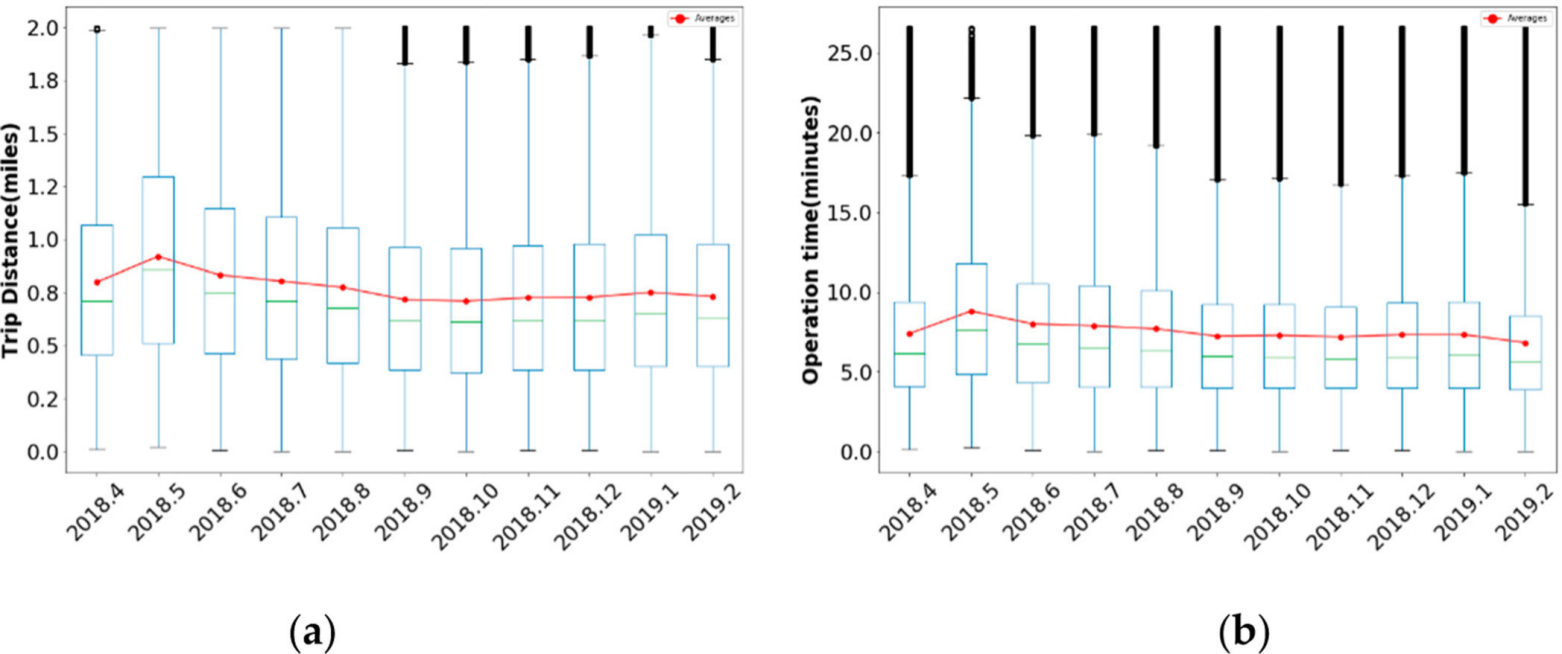
Boxplot analysis of monthly E-scooter trips in Austin TX. (**a**) Average E-scooter trip distance in different months; (**b**) Average E-scooter operation time in different months.

Figure 1 shows the average travel distance and operation time during each month. These trends were relatively stable, averaging around 0.77 miles and 7.55 min, respectively. The boxplot shows that the majority of E-scooters’ trips were shorter than two miles and were concentrated between the range of one half to one mile. Figure 1 also indicates that there were some longer outlier trips in each graph. These unusual trips were mechanically valid (i.e., travel range was within the maximum distance an E-scooter vehicle can cover); however, due to the absence of user information in the current dataset, we could not further explore their existence from a user’s aspect.

**Figure 2.**
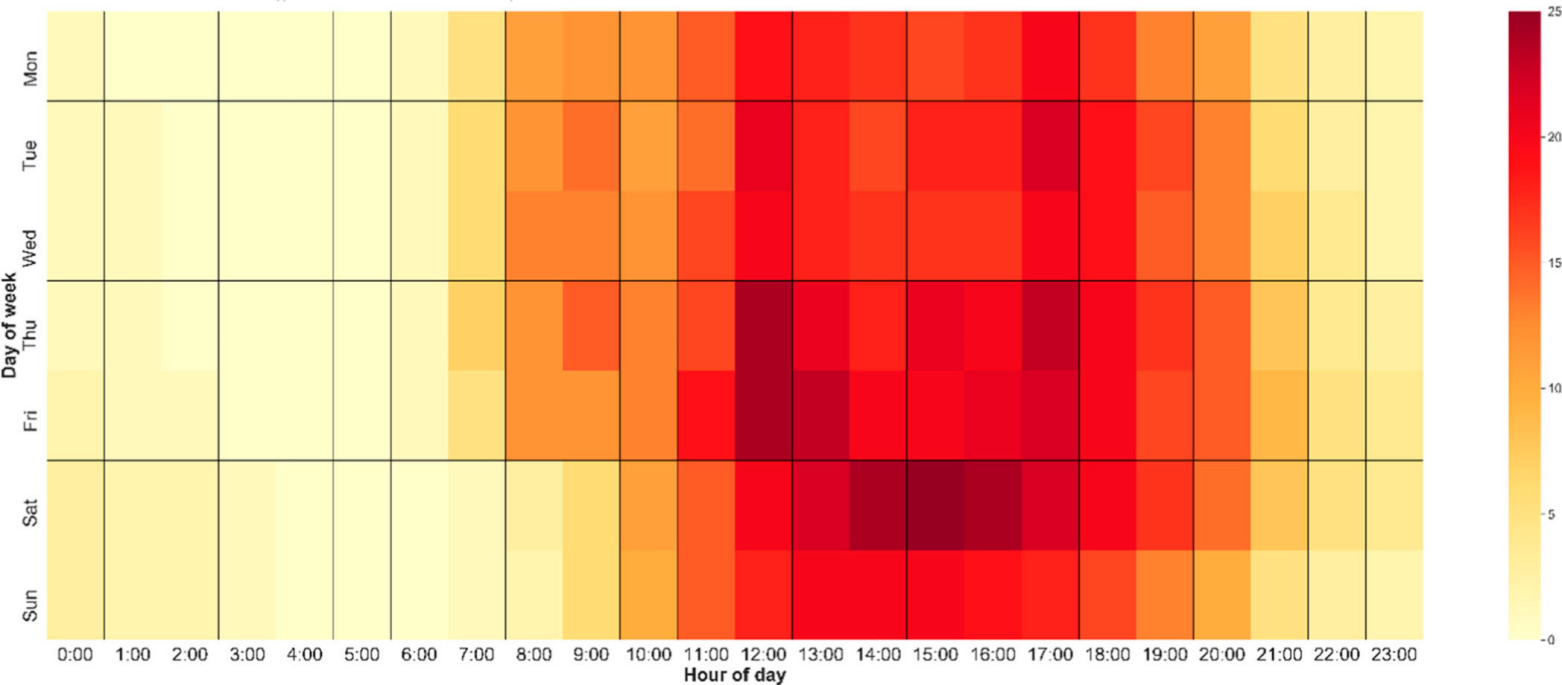
Heatmap of hourly E-scooter ridership (in thousands) in Austin, TX.

We also compared the E-scooter trips on weekdays with those on weekends. The results show that the average trip length, operation time, and average speed (assuming operation time equates to travel time) were 0.71 miles, 6.83 min, and 6.57 mph on weekdays, respectively. On weekends, passengers rode E-scooters significantly longer distances and durations (0.81 miles and 8.62 min), but at slower speeds (6.04 miles per hour). The difference between weekday and weekend travel patterns was significant under the 0.05 significance level. By calculating the hourly E-scooter usage counts, the authors were able to generate a heatmap of people’s daily E-scooter usage patterns (Figure 2). The heatmap showed that, on weekdays, riders tended to use E-scooters from 8 a.m. To 8 p.m. Hourly usage peaked at noon, 1 p.m., and 5 p.m. Comparatively, weekend riders often began their trips after 11 a.m., and usage remained high until the afternoon. After 8 p.m. on both weekdays and weekends, E-scooter ridership fell drastically.

### 3.3. Spatial Analysis

**Figure 3.**
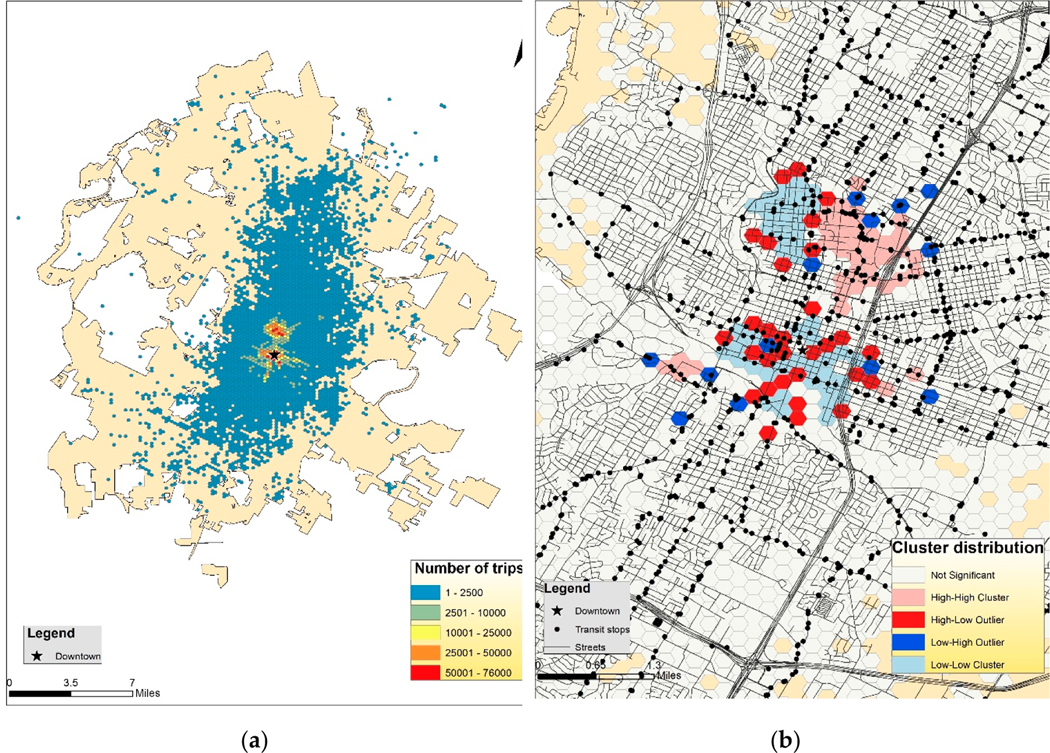
Spatial analysis of E-scooter usage in Austin TX. (**a**) E-scooter trip generation in Austin, TX; (**b**) Zoomed-in cluster analysis of E-scooter flow in Austin, TX.

Using the hexagons described in the data section above, we were able to generate an E-scooter usage spatial heatmap (Figure 3a). The number of trips indicated the summation of inflow and outflow trips related to each hexagonal area. From the figure, it is evident that most E-scooters trips were overwhelmingly clustered in two areas: Downtown Austin and the University of Texas at Austin (UT) campus (north of the downtown area). This suggested that college students were major E-scooter users. If we combine the temporal usage pattern with spatial visualization, it might indicate that the majority of E-scooter trips around UT Austin were generated by students commuting between school buildings to take classes.

To further understand the spatial distribution of E-scooter trips, the authors used Anselin Local Moran’s I in ArcGIS to model the spatial clustering of E-scooter trips (Figure 3b). In this map, the value of interest is the difference between inflow trips and outflow trips for each hexagon. Positive values represent significant E-scooter influx and negative values reflect otherwise. A significant positive Moran’s I index indicates that a feature is surrounded by similarly high or low neighboring features, whereas a significant negative index means the feature is an outlier because it is surrounded by dissimilar features. Based on these values, the cluster analysis distinguished four types of cluster and outliers: the clusters of high values (i.e., high-high cluster) and the opposite (i.e., low-low cluster), as well as the outliers in which a high value is predominantly encircled by low values (i.e., high-low outlier) and the opposite (i.e., low-high outlier). As shown in Figure 3b, there were no spatial clusters of E-scooter trips in Austin except Downtown and on UT’s campus, which means that E-scooter riders primarily started or ended their trips in these two areas. Specifically, there were many high-low clusters (dark red) in Downtown Austin, indicating that these areas experienced a significantly higher influx of E-scooter traffic. It is quite possible that these E-scooter hotspots show where riders frequently complete their trips. For UT, we saw two distinct zones (West Campus vs. Main Campus). The West Campus neighborhood was predominantly a low-low cluster (light blue), signifying a high number of E-scooter trips originated from here, whereas the Main Campus was a high-high cluster (pink), signifying a high number of trips were completed here. The inflow/outflow trip cluster further confirmed the presumption of the predominance of school commuting in E-scooter travel. Unfortunately, E-scooter travel patterns near downtown still remain uncertain.

### 3.4. E-scooter Usages and Surrounding Urban Environments

To better understand the impact of urban environments on E-scooter usage in Austin, the authors further modeled E-scooter usage with a negative binomial (NB) regression model. NB regression is widely used to model count data— ‘trip counts’ in this study. Compared to the Poisson regression model, the NB model handles over-dispersion more reliably (the variance of the dependent variable exceeding the mean by large), as is the case here. The maximum likelihood logarithmic function method was used to estimate the best fit utility function [28].

To investigate the overall E-scooter usage, we selected the total number of trips that either started or ended at each hexagon during the observation period as the dependent variable. The explanatory variables included demographic/socio-economic status (SES) and built environment (BE), two variable domains that are widely used in the study of urban environment and travel behavior [26,27]. A detailed list of the variables and corresponding basic statistics is provided in Table 2. To mitigate the impact of potential spatial autocorrelation, we calculated all scores at a fine resolution using the individual hexagon as the unit of analysis, which was 0.023 square mile in size and had an edge length of 500 ft. The small geographic unit warranted significant differences in terms of both the E-scooter usage and the built environments in neighboring units.

Five SES variables (population density, gender, age, income, and education) were included in the model. To measure these variables, the authors calculated the values at the scale of the Census Block Group (CBG) and assigned the CBG values to the corresponding hexagons. Population density referred to the number of people in thousands per square mile in each CBG. The gender variable was represented by the male-to-female ratio in the corresponding CBG. The age variable captured the young population within the geographic area by recording the proportion of those under 25 (upper bound of the youth defined by the United Nations [29]). The American Community Survey from the US Census categorized household income level into 17 groups, ranging from less than $10,000 per year to more than $200,000 per year. We retained the original categorization from the Census in this study and included all 17 income groups in the model. Finally, the education variable was measured using the proportion of people with a bachelor’s degree or higher in each CBG in order to capture the relationship between E-scooter usage and high educational background.

Eleven built environment variables were included in the model. The authors measured the Euclidean distances from the centroid of each hexagon to Austin city center and to the closest transit stop to measure proximity to the city center and accessibility to transit (buses, express buses, and light rail transit). To measure the street connectivity, the authors measured the number of cul-de-sacs and 4-way intersections within each hexagon. Besides these distance and connectivity tests, the authors also measured different land-use mix and percentages of each land use within each hexagon. The correlation test of built environment variables showed that all variables were independent of each other. Specifically, the land use mix was measured using a land-use entropy index [30]. The entropy index is an indicator from 0 to 1, in which larger values denote a more complex land use layout. A value of 0 was applied to the condition in which only one type of land use dominated the area.

1 $$ENT=\frac{-\left[\sum_{j=1}^{k}P^{j}*ln\left(P^{j}\right)\right]}{ln(k)}$$

where ENT is the land use entropy index; $P^{j}$ is the percentage of land use j within the study area; and k is the total number of land-use types within the study area,k≥2.

## 4. Results

### 4.1. Spatial-Temporal Patterns of E-scooter Usage in Austin

Descriptive analysis showed that, on average, there were more than 6000 monthly active E-scooters in Austin. They were used over 150,000 times per month on average and covered a total of over 110,000 miles. The average trip distance and duration was 0.77 miles and 7.55 min, respectively, and the average travel speed was 6.5 miles per hour. Within the study period, October 2018 was the peak month for E-scooter usage, with over 241,000 trips made by more than 7000 vehicles. The daily ridership analysis showed different usage patterns during weekdays and weekends. On weekdays, passengers rode E-scooters for significantly longer distances and durations than on weekends (0.81 miles vs. 0.71 miles; 8.62 min vs. 6.83 min). During the weekdays, passengers tended to use E-scooters from 8 a.m. To 8 p.m., and usage peaked around noon and 5 p.m. During the weekends, passengers tended to begin their trips after 11 a.m. and maintained a high usage throughout most of the afternoon. After 8 p.m. on both weekdays and weekends, ridership fell drastically.

To better reflect the spatial distribution of E-scooter usages in Austin while protecting users’ privacy, the Austin Transportation Department used hexagons to represent the origins and destinations of E-scooter trips. One hexagon covers roughly 0.023 square miles. There were approximately 12,000 hexagons across the city of Austin. Using these hexagons, the authors generated the E-scooter usage spatial heatmap (Figure 3a,b). From these figures, we can see that most E-scooters trips were overwhelmingly clustered in two areas: Downtown Austin and the University of Texas at Austin Campus.

Furthermore, the geospatial analysis showed that these two hotspots had distinct E-scooter usage characteristics. The majority of E-scooter usage in downtown Austin was outflow trips, with destinations near, but outside downtown. Similar trends were also found west of the UT campus, a location densely populated with student housing units, implying that students would use E-scooters to travel from their residences to outside destinations. Contrary to the mentioned areas, trips data from the UT campus indicated that campus was primarily a destination, making it an inflow hub.

The empirical results are rather intuitive. First, these three areas are well equipped to support E-scooter travels with both solid bike and pedestrian infrastructure and street connectivity. Second, demand for E-scooters in these areas was presumably much higher. Downtown riders perhaps use E-scooters to commute to work or travel to transit stops, while tourists might use E-scooters for sightseeing and moving around the city. For both the West and Main campus, students seem to use E-scooters to travel from their residences to academic buildings or within separate academic buildings on campus.

### 4.2. E-scooter Usage and Surrounding Urban Environments

This paper used a negative binomial regression model to explore the relationship between E-scooter usage within each hexagon and the surrounding built environments. STATA 15.0 software was used to build the statistical model. The unit of analysis was the hexagon defined by the Austin Transportation Department. The final model showed that 15 variables were significantly associated with E-scooter usage, five of which were negatively correlated (Table 3).

For all SES variables, higher population density, more males, and more residents with higher education were correlated with more E-scooter usage, whereas the average household income level held a negative relationship. The results coincide with the user survey report published by the city of Austin and Lime, which stated that most trips were generated in the city’s populous regions, by a majority of male users, by middle or low-income groups, and by those who are highly educated [25]. Surprisingly, the proportion of the young population within each hexagon was negatively correlated with E-scooter usage, which contradicted the assumption that the young population mainly used E-scooters. Lime also reported similar findings that the global average age of E-scooter riders in 2018 was 32, and over one-quarter of them were older than 37 [31].

As for our built environment variables, the model revealed that the further the distance from the city center and/or a transit stop, the less likely E-scooter trips were to take place (1 mile further away from the city center (transit) will cause approximately 33% (62%) ridership decrease). Better street connectivity (i.e., fewer dead ends, more 4-way intersections) could potentially experience more ridership, although the percentage change was small. Compact land use tended to associate with increased E-scooter trips (one unit increase in the land-use entropy index would relate to double ridership). Surprisingly, the proportion of residential areas within each hexagon did not show a significant correlation to the number of E-scooter trips. As for land use types, the percentage of mixed-use, educational, open space, commercial, and transit facility areas within each hexagon were all positively correlated with E-scooter usage. In particular, a one-percent increase in the mixed land use could relate to an over 50% increase in ridership, making it the most relevant land use. However, the percentage of commercial and transit facilities’ land use within each hexagon were only significant at a less-restricted level with small coefficients, implying that the presence of commercial facilities and transit connections had a modest impact on E-scooter usage.

## 5. Conclusions and Discussions

This paper analyzed the 1.7 million E-scooters trips from April 2018 to February 2019 in Austin, TX. The results show, on average, that were over 6000 monthly active E-scooters, generating 150,000 trips and traveling 117,000 miles. The average travel distance and time were 0.77 miles and 7.55 min. This paper further identified two E-scooter usage hotspots in Downtown Austin and UT Campus. Specifically, Downtown Austin was mainly an origination point, where many trips were generated. The Main UT campus was a destination, attracting a higher proportion of inflow trips. Due to the high concentration of student housing units, West UT campus also generated many E-scooter trips that possibly ended at the Main Campus.

The negative binomial model showed that hexagons with high population density, lower household income, more males, and higher education were more likely to generate E-scooter trips. Surprisingly, the percentage of young people (under 25) within each hexagon was negatively associated with total E-scooter trips. For built environment variables, the model confirmed that hexagons near the city center or transit stops were positively correlated to E-scooter usage. Better street connectivity and more complex land use also related to greater E-scooter traffic. Among all the land use types tested in the model, the most influential variables were the degree of land use mix, followed by the percentage of the educational area and open space within each hexagon. The percentages of commercial area and transit facilities were only marginally significant in the model, while the percentage of residential areas was not significant in the model. However, a limitation of the model is that the potential spatial autocorrelation could hinder the interpretation of the exact margins of these variables. Nevertheless, the model results identify some key factors that were beneficial to biking and scootering in previous studies.

We would argue that the E-scooter is a new transportation mode between walking and biking. It covers the travel demand gap between walking and biking in cases where a trip is both too long to walk, but also too short to ride a bike. However, how to manage and regulate this new mode has become a problem for many US cities. As a progressive city, Austin has published several city-level operation rules for both private operators and riders. However, the problems still exist. For example, according to the Austin 311 call website (non-emergency calls to report complaints of public services), there were 3145 E-scooter complaints (e.g., sidewalk and public space obstruction) from March to August 2019 [32]. We believe that a lack of infrastructure dedicated to E-scooters increased the difficulty in regulating their operation.

We also argue that E-scooters are closely related to dockless bikes. Both of them focus on people’s micro-mobility needs and rely on similar dockless and mobile payment technologies. This paper further proved the similarity between their travel behaviors and the surrounding environments. Thus, it might be possible to apply some cyclist rules to E-scooter users. Further, in many cities, the shared mobility market has either fizzled out or reached equilibrium. Many docked or dockless bike program’s previous popularity no longer exists [33]. Many industry frontrunners (e.g., OFO in China, Pronto in Seattle, US) have faced devastating consequences from reckless expansion and haphazard management [33,34]. Thus, we believe that by studying E-scooter travels, we could better regulate the current and future shared micro-mobility market.

The results of this study coincide with previous studies and reaffirm the close connection between E-scooter usage and some key built environment indicators such as downtown, university, connected streets, and mixed land use [11,12,21,22]. Noticeably, nevertheless, the cities in these studies are either Chinese cities or progressive cities such as Austin or Seattle in the United States, where people would love to see and use bikes or scooters to replace car vehicles. Therefore, the generalizable results could only apply to cities where people already embrace or start to embrace the idea of shared mobility. It is beyond the scope of this study to claim a shift in people’s travel behavior because of the emerging shared mobility.

Traditional survey-based transportation data analyses are often constrained by data collection cost, survey bias, and small sample size [35]. In this paper, we utilized big data analytic techniques and revealed important E-scooter travel patterns from a massive dataset. As exploratory research, this study verified the capability of using big data to analyze new transportation modes when a traditional survey is not available. The future of shared mobility research would benefit from the development of urban informatics and big data that could extract detailed travel information from massive datasets that are detached from a demographic and socio-economic representation of the space.

## Figures and Tables

**Table 1.** Descriptive Statistics of E-scooter Trip Features by Month in Austin.

Months	Trip Counts	# Operational Vehicles	Total Mileage Traveled (mi)	Avg Trip Distance (mi)	Avg Operation Time (min)
Apr 2018	36,710	1180	29,328	0.80	7.40
May 2018	5190	370	4777	0.92	8.82
Jun 2018	48,150	1230	40,111	0.83	8.02
Jul 2018	82,610	1690	66,414	0.80	7.89
Aug 2018	206,680	3440	160,140	0.77	7.70
Sep 2018	229,050	6360	164,299	0.72	7.24
Oct 2018	241,650	7350	171,610	0.71	7.29
Nov 2018	236,210	12,010	171,503	0.73	7.19
Dec 2018	214,660	12,150	156,243	0.73	7.33
Jan 2019	192,570	10,200	144,667	0.75	7.34
Feb 2019	246,810	11,150	180,733	0.73	6.83
Average	158,208	6103	117,257	0.77	7.55

**Table 2.** Descriptive statistics of dependent (DV) and independent variable (IV) for the NB model.

Name	Type	Unit	Mean	Sd	Min	Max	Count
Number of trips (DV)	Numeric	Count	846	4808	1	75,951	3898
Population density (IV)	Numeric	1000 people/sq. mi	5.12	4.13	0	53.35	3898
Gender (IV)	Categorical	Male/female	1.10	0.34	0.43	4.87	3898
Age (IV)	Numeric	%	28.55	13.16	0	98	3898
Income level (IV)	Categorical	17 Categories	9	2	0	14	3898
Education (IV)	Numeric	%	50.07	24.17	0	99	3898
Distance to city center (IV)	Numeric	mile	4.80	2.69	0.07	13.67	3898
Distance to transit (IV)	Numeric	mile	0.25	0.39	0	7.18	3898
# of Cul-de-sac (IV)	Numeric	Count	48	20	2	135	3898
# of 4-way intersection (IV)	Numeric	Count	84	69	0	371	3898
Land use mix (IV)	Numeric	Entropy Value	0.70	0.09	0	0.92	3898
Residential area (IV)	Numeric	%	48.65	15.62	0	81	3898
Commercial area (IV)	Numeric	%	9.06	6.60	0	30	3898
Mixed-use area (IV)	Numeric	%	0.57	1.04	0	5	3898
Educational area (IV)	Numeric	%	3.98	3.47	0	17	3898
Open space and parks (IV)	Numeric	%	12.99	11.53	0	100	3898
Transit facility (IV)	Numeric	%	0.41	0.75	0	4	3898

**Table 3.** Final Model Output for the E-scooter Trip Analysis in Austin, TX.

Explanatory Variable	Coefficient	Std. Error	Z-Score
Population density	0.101	0.01	15.18 **
Gender (Male)	0.430	0.06	7.19 **
Age	−0.009	0.00	−4.65 **
Income level	−0.145	0.02	−8.46 **
Education	0.020	0.00	14.32 **
Distance to city center	−0.327	0.01	−32.19 **
Distance to transit	−0.623	0.07	−9.05 **
# of Cul-de-sac	−0.013	0.00	−9.97 **
# of 4-way intersection	0.006	0.00	12.48 **
Land use mix	2.225	0.37	6.08 **
Residential area	0.001	0.00	0.42
Commercial area	0.007	0.00	1.81 *
Mixed-use area	0.548	0.03	17.52 **
Educational area	0.062	0.01	8.8 **
Open space and parks	0.018	0.00	6.88 **
Transit facility	0.066	0.03	1.92 *
Constant	3.120	0.39	7.94 **

Note:

** significant at 0.05 level

* significant at 0.10 level.
